# Impact of splenic hilar lymph node metastasis on prognosis in patients with advanced gastric cancer

**DOI:** 10.18632/oncotarget.18762

**Published:** 2017-06-28

**Authors:** Taeil Son, In Gyu Kwon, Joong Ho Lee, Youn Young Choi, Hyoung-Il Kim, Jae-Ho Cheong, Sung Hoon Noh, Woo Jin Hyung

**Affiliations:** ^1^ Department of Surgery, Yonsei University College of Medicine, Seoul, South Korea; ^2^ Gastric Cancer Center, Yonsei Cancer Center, Seoul, South Korea; ^3^ Department of Surgery, Keimyung University School of Medicine, Daegu, South Korea; ^4^ Department of Surgery, National Health Insurance Service Ilsan Hospital, Goyang, South Korea

**Keywords:** gastric cancer, D2 lymphadenectomy, splenic hilar lymph node, prognosis

## Abstract

**Background:**

Impact of splenic hilar LN dissection during total gastrectomy for proximal advanced gastric cancer is controversial. The objective of this study was to assess the impact on prognosis of splenic hilar lymph node(LN) metastasis compared to that of metastasis to other regional LN groups.

**Study Design:**

Patients who underwent total gastrectomy with D2 LN dissection from 2000 to 2010 were reviewed retrospectively. The clinicopathologic characteristics and long-term results of patients with splenic hilar LN metastasis were compared to those of patients with only metastasis to other extraperigastric LNs (stations #8a, #9, #11, or #12a). To investigate the survival benefit of performing splenic hilar LN dissection, the estimated therapeutic index for the procedure was calculated by multiplying the incidence of metastases in the hilar region by the survival rates for individuals with nodal involvement in that region.

**Results:**

Of 602 patients, 87(14.5%) had hilar LN metastasis. The 5-year overall and relapse-free survival rates for patients with hilar LN metastasis were 24.1% and 12.1%, respectively. These rates were similar to those for patients with metastasis to other extraperigastric LNs (*P* > 0.05), with similar recurrence patterns. Overall survival in the hilar LN metastasis group was better than that for patients with distant metastasis(*P* < 0.05). The estimated therapeutic index of splenic hilar LN dissection was 3.5, which was similar to index values for LN dissection at other extraperigastric LNs.

**Conclusions:**

Dissection of splenic hilar LNs during total gastrectomy for advanced gastric cancer allows for a prognosis similar to that achieved with dissection of extraperigastric LNs.

## INTRODUCTION

Gastric cancer is the fifth most common malignancy and the third leading cause of cancer death in the world [1]. Most gastric cancer is initially diagnosed at the advanced disease stage except in countries that have mass-screening programs [1–5]. Although the incidence of gastric cancer worldwide has remained steady, increases in proximal gastric cancers, including esophagogastric junction cancer [6], have bolstered recommendations for performing total gastrectomy with D2 lymph node (LN) dissection for proximal advanced gastric cancer [7–9].

The reported incidence of metastasis to splenic hilar LNs in proximal gastric cancer ranges from 5.8 to 26.7% [10–16]. Thus, most treatment guidelines for proximal gastric cancer recommend dissection of the splenic hilar LNs as a regional LN group during total gastrectomy with D2 LN dissection [7–9]. To accomplish this dissection, surgeons typically perform either a splenectomy or a spleen-preserving technique. Although splenectomies have been found to be associated with high morbidity and mortality, spleen-preserving hilar LN dissection can be technically demanding [15–18], and therefore surgeons are reluctant to perform splenic hilar LN dissection during total gastrectomy for proximal advanced gastric cancer. More importantly, the prognostic impact of metastasis to the splenic hilar LNs has yet to be determined, obscuring the necessity of performing splenic hilar LN dissection during gastrectomy.

The aim of this study was to assess the impact of splenic hilar LN metastasis on prognosis in gastric cancer patients via comparison of the recurrence patterns and overall and relapse-free survival rates in cases of splenic hilar LN metastasis with those in cases of metastasis to other regional LNs. We also compared overall survival for splenic hilar LN metastasis with that for distant metastasis. Lastly, we calculated and compared estimated therapeutic index values for splenic hilar LN dissection and dissection of other regional LNs.

## RESULTS

### Patient demographics

Of the 602 patients who underwent total gastrectomy with D2 LN dissection, 258 patients received a splenectomy, and 344 patients received splenic hilar LN dissection without splenectomy. Among gastrectomy patients, 87 (14.5%) had hilar LN metastasis, and 515 (85.5%) had no hilar LN metastasis. The mean age of the LN #10-positive group versus the LN #10-negative group was 54.9 y versus 56.6 y (*p* = 0.225), with 52 (59.8%) versus 351 (68.2%) male patients (*p* = 0.124) in each group, respectively. Tumors from LN #10-positive patients showed undifferentiated histology more frequently (*p* = 0.002), were of larger size (*p* < 0.001), were more frequently found in the greater curvature of the stomach or as an encircling lesion (*p* < 0.001), comprised more Borrmann type IV cancers (*p* < 0.001), and exhibited more frequent lympho-vascular involvement (*p* < 0.001) than tumors from LN #10-negative patients. Although the mean number of retrieved LNs in the LN #10-positive group did not differ from that in the LN #10-negative group (56.1 vs. 52.3, *p* = 0.063), the mean number of metastatic LNs was significantly greater (25.5 vs. 7.0, *p* < 0.001) in the LN #10-positive group. As expected, LN #10-positive patients had more advanced T, N, and TNM stage cancers than LN #10-negative patients (*p* < 0.001 for all comparisons; Table 1).

**Table 1:** Clinicopathologic factors for patients classified according to metastasis to lymph node station 10

Variable	LN#10 (+) (*n* = 87)			LN#10 (−) (*n* = 515)			P*
	No. of Patients		%	No. of Patients		%	
Age, years							0.225
Mean		54.9			56.6		
SD		13.1			12.2		
Sex							0.124
Male	52		59.8	351		68.2	
Female	35		40.2	164		31.8	
Splenectomy							< 0.001
Yes	56		64.4	204		39.6	
No	31		35.6	311		60.4	
Operation procedure							0.407
Open	85		97.7	489		95.0	
Minimally invasive	2		2.3	26		5.0	
Histologic type							0.002
Differentiated	10		11.5	137		26.6	
Undifferentiated	77		88.5	378		73.4	
Tumor size, cm							< 0.001
Mean		87.1			64.2		
SD		43.1			33.8		
Location							< 0.001
LC	28		32.2	260		50.5	
GC	19		21.8	47		9.1	
AW	14		16.1	76		14.8	
PW	17		19.5	121		23.5	
Circular	9		10.4	11		2.1	
Gross type							< 0.001
Borrmann I	4		4.6	34		6.6	
Borrmann II	8		9.2	116		22.5	
Borrmann III	38		43.7	252		48.9	
Borrmann IV	35		40.2	108		21.0	
Borrmann V	2		2.3	5		1.0	
LV invasion							< 0.001
negative	7		8.0	195		37.9	
positive	80		92.0	320		62.1	
Number of metastatic LNs							< 0.001
Mean		25.5			7.0		
SD		18			10.2		
Number of retrieved LNs							0.063
Mean		56.1			52.3		
SD		17.7			17.9		
T classification							< 0.001
T2	2		2.3	62		12.0	
T3	6		6.9	121		23.5	
T4a	67		77.0	317		61.6	
T4b	12		13.8	15		2.9	
N classification							< 0.001
N0	0		0.0	143		27.8	
N1	2		2.3	85		16.5	
N2	8		9.1	107		20.8	
N3	77		88.5	180		35.0	
TNM stage							< 0.001
Stage IB	0		0.0	37		7.2	
Stage IIA	0		0.0	55		10.7	
Stage IIB	3		3.5	88		17.1	
Stage IIIA	0		0.0	84		16.3	
Stage IIIB	11		12.6	101		19.6	
Stage IIIC	73		83.9	150		29.1	

Abbreviations: LN, lymph node; SD, standard deviation; LC, lesser curvature; GC, greater curvature; AW, anterior wall; PW, posterior wall; LV, lympho-vascular. *P-values were determined for Student's *t*-test or χ^2^ (Fisher's exact) test for continuous or categorical factors, respectively.

Clinicopathologic characteristics were compared between groups with metastasis to other extraperigastric LNs stations (stations #8a, #9, #11, or #12a) but not to the splenic hilar LNs, and subgroups of patients with metastasis to the hilar LN #10 but not to each of the other stations. In this comparison, there was no patient with metastasis to LN #10 but not to other extraperigastric LN. The LN #10-positive subgroups had more advanced pathologic T classifications than any extraperigastric, LN #8a-, LN #9-, and LN #12a-positive groups (*p* < 0.001, 0.011, 0.037, and < 0.001, respectively). The number of metastatic LNs and N classifications of the LN #10-positive subgroups, however, were similar to those for the LN #8a-, LN #9-, LN #11-, and LN #12a-positive groups. Overall, the LN #10-positive subgroup patients showed more advanced stage disease than any extraperigastric, LN #8a-, LN #9-, and LN #12a-positive patients but not LN #11-positive patients (Table 2).

**Table 2:** Clinicopathologic factors of patients with metastasis to lymph node station 10 in comparison to patients with exclusive metastasis to other extraperigastric LN stations

Variable	LN#10(+) (*n* = 87)	LN#10(−) any *N*2(+) (*n* = 123)		LN#10(+) LN#8a(−) (*n* = 54)	LN#10(−) LN#8a(+) (*n* = 51)		LN#10(+) LN#9(−) (*n* = 57)	LN#10(−) LN#9 (+) (*n* = 67)		LN#10(+) LN#11(−) (*n* = 53)	LN#10(−) LN#11(+) (*n* = 44)		LN#10(+) LN#12a(−) (*n* = 85)	LN#10 (−) LN#12a (+) (*n* = 14)	
	No. of Patients (%)	No. of Patients (%)	P*	No. of Patients (%)	No. of Patients (%)	P*	No. of Patients (%)	No. of Patients (%)	P*	No. of Patients (%)	No. of Patients (%)	P*	No. of Patients (%)	No. of Patients (%)	P*
Histologic type			0.031			0.056			0.142			> 0.999			< 0.001
Differentiated	10 (11.5)	29 (23.6)		6 (11.1)	13 (25.5)		7 (12.3)	15 (22.4)		3 (5.7)	3 (6.8)		9 (10.6)	8 (57.1)	
Undifferentiated	77 (88.5)	94 (76.4)		48 (88.9)	38 (74.5)		50 (87.7)	52 (77.6)		50 (94.3)	41 (93.2)		76 (89.4)	6 (42.9)	
Tumor size, mm			0.014			0.002			0.422			0.662			0.298
Mean	87.1	73.1		86.4	65.4		81.7	75.9		80.3	77.2		87.2	74.6	
SD	43.1	35.9		41.5	23.8		39.3	41.4		38.7	30.0		43.3	29.9	
Location			0.003			0.002			0.048			0.101			0.569
LC	28 (32.2)	67 (54.5)		17 (31.5)	32 (62.7)		19 (33.3)	35 (52.2)		16 (30.2)	23 (52.3)		27 (31.8)	6 (42.9)	
GC	19 (21.8)	12 (9.8)		13 (24.1)	3 (5.9)		12 (21.1)	11 (16.4)		14 (26.4)	4 (9.1)		19 (22.3)	1 (7.1)	
AW	14 (16.1)	18 (14.6)		9 (16.7)	6 (11.8)		10 (17.5)	12 (17.9)		11 (20.8)	6 (13.6)		14 (16.5)	4 (28.6)	
PW	17 (19.6)	23 (18.7)		10 (18.5)	10 (19.6)		11 (19.3)	9 (13.4)		8 (15.1)	8 (18.2)		16 (18.8)	2 (14.3)	
Circular	9 (10.3)	3 (2.4)		5 (9.2)	0 (0)		5 (8.8)	0 (0)		4 (7.5)	3 (6.8)		9 (10.6)	1 (7.1)	
Number of metastatic LN			< 0.001			0.207			0.125			0.716			0.135
Mean	25.5	15.7		20.8	17.0		23.3	18.7		20.6	19.4		25.2	17.4	
SD	18	13.3		15.4	15.4		17.9	15.4		15.8	16.3		17.4	12	
Number of retrieved LN			0.309			0.515			0.763			0.775			0.085
Mean	56.1	53.5		54.9	52.7		56.4	55.4		51.5	52.5		55.8	47.2	
SD	17.7	19		16.6	17.1		16.8	22.3		15.7	16.3		17.2	16.4	
T classification			< 0.001			0.011			0.037			0.553			< 0.001
T2	2 (2.3)	5 (4.1)		2 (3.7)	3 (5.9)		2 (3.5)	2 (3.0)		2 (3.8)	1 (2.3)		2 (2.4)	0 (0)	
T3	6 (6.9)	24 (19.5)		2 (3.7)	7 (13.7)		4 (7.0)	14 (20.9)		4 (7.5)	3 (6.8)		5 (5.9)	7 (50.0)	
T4a	67 (77.0)	92 (74.8)		43 (79.6)	41 (80.4)		44 (77.2)	49 (73.1)		40 (75.5)	38 (86.4)		66 (77.6)	7 (50.0)	
T4b	12 (13.8)	2 (1.6)		7 (13.0)	0 (0)		7 (12.3)	2 (3.0)		7 (13.2)	2 (4.5)		12 (14.1)	0 (0)	
N classification			0.065			0.583			0.491			0.809			> 0.999
N1	2 (2.3)	6 (4.9)		2 (3.7)	1 (1.9)		2 (3.5)	3 (4.5)		1 (1.9)	2 (4.5)		2 (2.4)	0 (0)	
N2	8 (9.2)	24 (19.5)		8 (14.8)	11 (21.6)		6 (10.5)	12 (17.9)		7 (13.2)	5 (11.4)		8 (9.4)	1 (7.1)	
N3	77 (88.5)	91 (75.6)		44 (81.5)	39 (76.5)		49 (86.0)	52 (77.6)		45 (84.9)	37 (84.1)		75 (88.2)	13 (92.9)	
TNM stage			0.001			0.059			0.013			0.237			0.001
Stage IIA	0 (0)	1 (0.8)		0 (0)	1 (2.0)		0 (0)	0 (0)		0 (0)	0 (0)		0 (0)	0 (0)	
Stage IIB	3 (3.5)	4 (3.2)		3 (5.5)	1 (2.0)		3 (5.3)	2 (3.0)		3 (5.7)	1 (2.3)		3 (3.5)	0 (0)	
Stage IIIA	0 (0)	13 (10.6)		0 (0)	4 (7.8)		0 (0)	8 (11.9)		0 (0)	3 (6.8)		0 (0)	0 (0)	
Stage IIIB	11 (12.6)	27 (22.0)		7 (13.0)	11 (21.6)		7 (12.3)	13 (19.4)		7 (13.2)	4 (9.1)		10 (11.8)	8 (57.1)	
Stage IIIC	73 (93.9)	78 (63.4)		44 (81.5)	34 (66.6)		47 (82.4)	44 (65.7)		43 (81.1)	36 (81.8)		72 (84.7)	6 (42.9)	

Abbreviations: LN, lymph node; SD, standard deviation; LC, lesser curvature; GC, greater curvature; AW, anterior wall; PW, posterior wall; LV, lympho-vascular. *P-values were calculated from Student's *t*-test or χ^2^ (Fisher's exact) test for continuous or categorical factors, respectively.

### Oncologic outcomes and survival analyses

**Figure 1:**
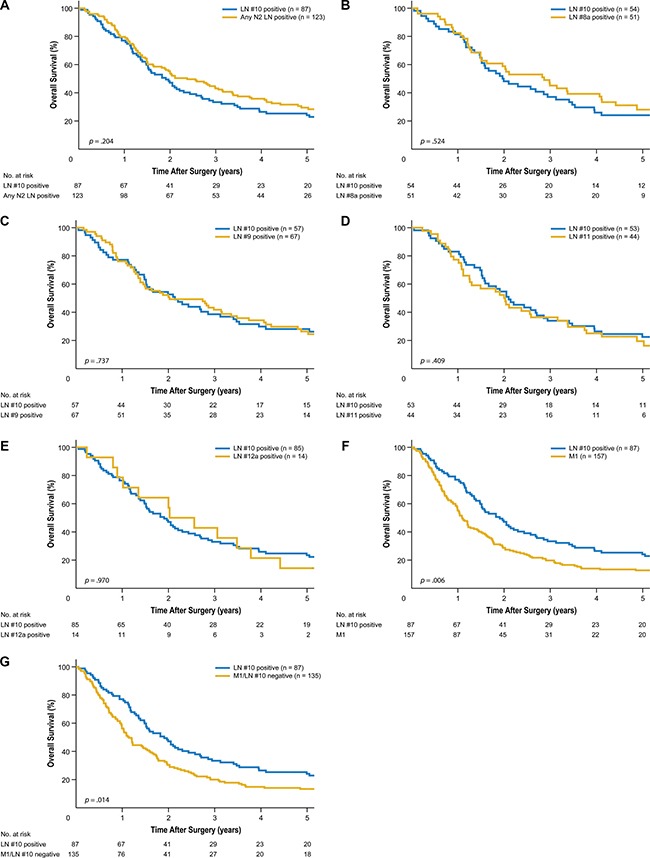
Kaplan-Meier overall survival curves for patients with splenic hilar lymph node (LN) metastasis compared to those with metastasis to extraperigastric LNs (**A**) Compared to metastasis to any extraperigastric LN (*P* = 0.204). (**B**) Compared to LN #8a metastasis (*P* = 0.524). (**C**) Compared to LN #9 metastasis (*P* = 0.737). (**D**) Compared to LN #11 metastasis (*P* = 0.409). (**E**) Compared to LN #12a metastasis. (*P* = 0.970). (**F**) Compared to distant metastasis (M1) (*P* = 0.006). (**G**) Compared to M1 excluding LN #10 metastasis (*P* = 0.014).

Long-term follow-up over a median of 89 months revealed significant differences in overall survival between LN #10-positive patients and LN #10-negative patients (5-year overall survival of 24.1% vs. 54.8%, respectively, *p* < 0.001). Meanwhile, no significant differences in overall survival were found between patients in the LN #10 subgroups and those with metastasis to LN #8a, LN #9, LN #11, or LN #12a (5-year overall survival of 24.1% vs. 28.0%, *p* = 0.524; 28.1% vs. 26.4%, *p* = 0.737; 22.5% vs. 19.5%, *p* = 0.409; and 23.5% vs. 14.3%, *p* = 0.970, respectively). Overall survival in the LN #10-positive group was, however, better than that of LN #10-positive patients with distant metastasis (M1) and that of patients with distant metastasis but no LN #10 metastasis (*p* = 0.006 and *p* = 0.014, respectively; Figure 1).

**Figure 2:**
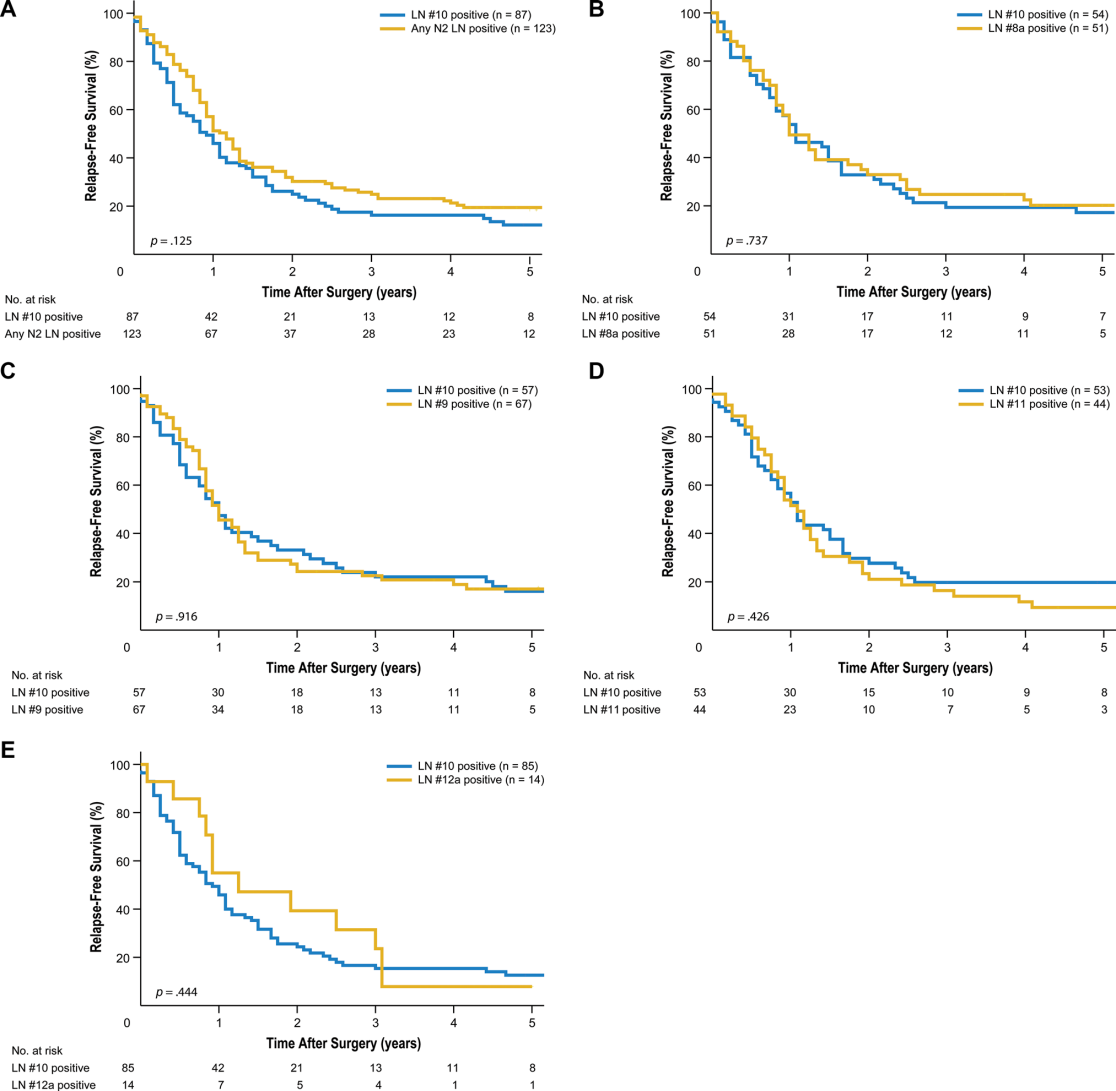
Kaplan-Meier relapse-free survival curves for patients with splenic hilar lymph node (LN) metastasis compared to those with metastasis to extraperigastric LNs (**A**) Compared to metastasis to any extraperigastric LN (*P* = 0.125). (**B**) Compared to LN #8a metastasis (*P* = 0.737). (**C**) Compared to LN #9 metastasis (*P* = 0.916). (**D**) Compared to LN #11 metastasis (*P* = 0.426). (**E**) Compared to LN #12a metastasis (*P* = 0.444).

With regard to relapse-free survival, LN #10-positive subgroup patients experienced survival similar to that in LN #8a-, LN #9-, LN #11-, and LN #12a-positive patients (5-year relapse-free survival of 17.2% vs. 20.2%, *p* = 0.737; 16.0% vs. 17.0%, *p* = 0.916; 19.8% vs. 9.4%, *p* = 0.426; and 12.6% vs. 7.9%, *p* = 0.444, respectively; Figure 2). Recurrence patterns in LN #10-positive patients were also similar to those in LN #8a-, LN #9-, LN #11-, and LN #12a-positive patients (*p* = 0.596, 0.134, 0.712, and 0.085, respectively; Table 3). The most common pattern of recurrence was peritoneal recurrence, regardless of involvement at any other extraperigastric LN station.

**Table 3:** Recurrence patterns

Groups	Locoregional	Hematogenous	Distant LN	Peritoneal	Mixed	*P*
LN#10+ (*n* = 64)	3 (4.7)*	8 (12.5)	4 (6.3)	37 (57.8)	12 (18.8)	0.389
LN#10− (*n* = 212)	5 (2.4)	36 (17.0)	21 (9.9)	125 (59.0)	25 (11.8)	
LN#10+ (*n* = 64)	3 (4.7)	8 (12.5)	4 (6.3)	37 (57.8)	12 (18.8)	0.506
LN#10-AnyN2+ (*n* = 87)	2 (2.3)	13 (14.9)	12 (13.8)	48 (55.2)	12 (13.8)	
LN#10+LN#8− (*n* = 37)	2 (5.4)	4 (10.8)	2 (5.4)	22 (59.5)	7 (18.9)	0.596
LN#10-LN#8+ (*n* = 36)	1 (2.8)	5 (13.9)	6 (16.7)	19 (52.8)	5 (13.9)	
LN#10+LN#9− (*n* = 41)	3 (7.3)	7 (17.1)	2 (4.9)	22 (53.7)	7 (17.1)	0.134
LN#10−LN#9+ (*n* = 50)	0 (0)	10 (20.0)	9 (18.0)	24 (48.0)	7 (14.0)	
LN#10+LN#11− (*n* = 36)	0 (0)	5 (13.9)	3 (8.3)	23 (63.9)	5 (13.9)	0.712
LN#10-LN#11+ (*n* = 37)	1 (2.7)	3 (8.1)	6 (16.2)	22 (59.5)	5 (13.5)	
LN#10+LN#12a− (*n* = 62)	3 (4.8)	7 (11.3)	4 (6.5)	37 (59.7)	11 (17.7)	0.085
LN#10−LN#12a+ (*n* = 11)	0 (0)	3 (27.3)	0 (0)	3 (27.3)	5 (45.5)	

*Values in parentheses are percentages.

### Therapeutic index of lymph node dissection at each lymph node station after total gastrectomy with D2 lymph node dissection

**Figure 3:**
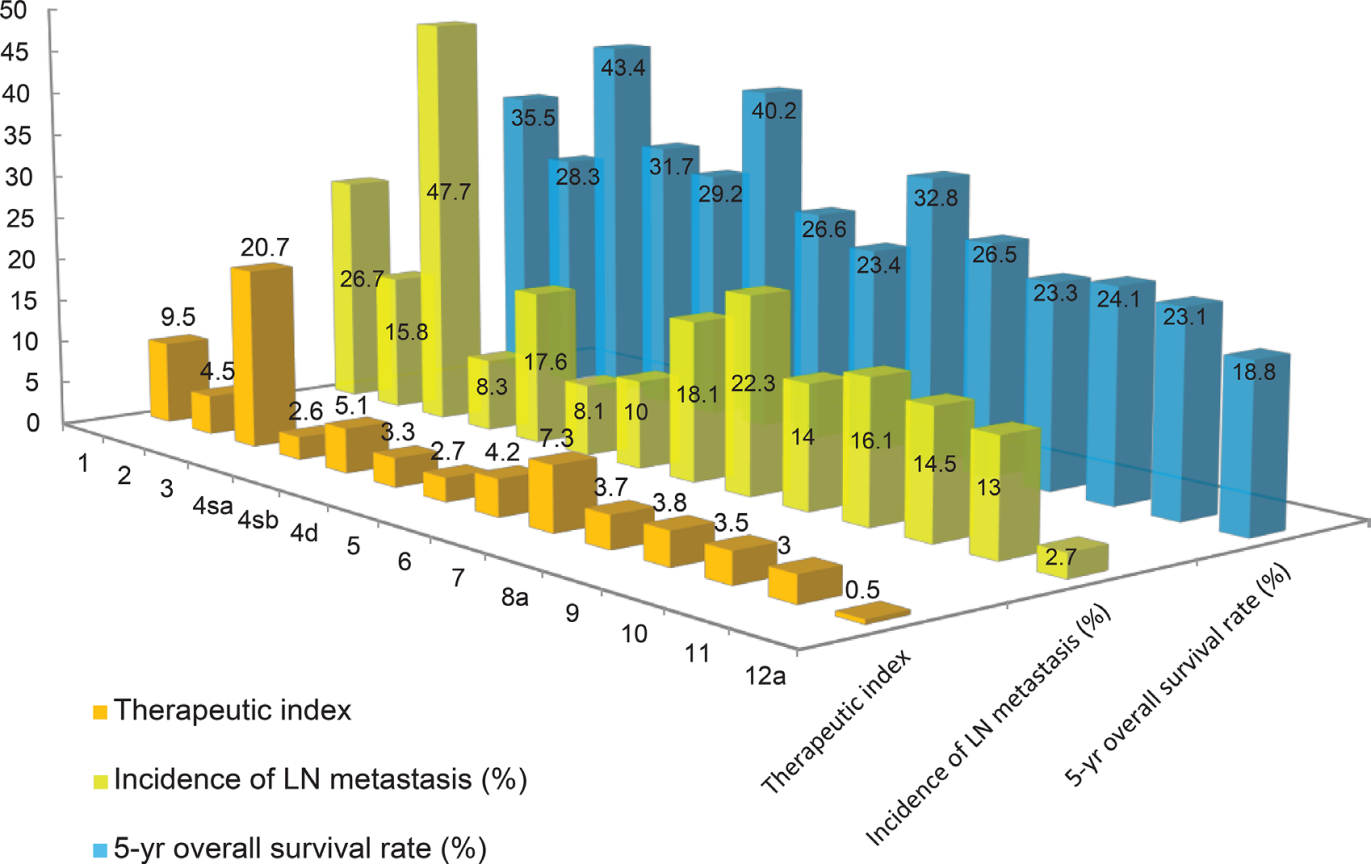
Estimated benefit of lymph node (LN) dissection by calculating therapeutic index Therapeutic index was calculated by multiplying the frequency of metastases by 5-year survival rates for individuals with nodal involvement at that specific station, regardless of nodal metastases to any other LN station.

The incidences of metastasis and the 5-year overall survival rates for each LN station are provided in Figure 3. The incidences of LN metastasis at LN #8a, LN #9, LN #11, and LN #12a were 14.0%, 16.1%, 13.0%, and 2.7%, respectively. The incidence of metastasis to the splenic hilar LNs was 14.5%. The therapeutic index values of the estimated benefit of dissection of perigastric LN stations (stations #1 to #7) ranged from 2.6 to 20.7. The therapeutic index values for dissection of extraperigastric LNs (stations #8a, #9, #11, and #12a) ranged from 0.5 to 3.8. The estimated therapeutic value of splenic hilar LN dissection was 3.5 and was calculated as follows: [0.145 (incidence of splenic hilar LN metastasis) × 24.1 (5-year overall survival)]. Thus, the therapeutic index value of splenic hilar LN dissection was within the range of therapeutic index values for dissection of extraperigastric LN stations.

## DISCUSSION

The prognosis for advanced gastric cancer patients with splenic hilar LN metastasis in the current study was similar to that for patients with any extraperigastric LN metastasis and better than that for those with distant metastasis. Relapse-free survival and patterns of recurrence in the patients with splenic hilar LN metastasis were also comparable to patients with other extraperigastric LN metastasis. Additionally, calculation of the therapeutic index of splenic hilar LN dissection during total gastrectomy for the treatment of proximal advanced gastric cancer revealed that the procedure had an impact on patient survival similar to dissection of other extraperigastric LNs.

Although current treatment guidelines recommend splenic hilar LN dissection during total gastrectomy with D2 LN dissection, the prognosis of patients with splenic hilar LN metastasis is poor. The discovery that hilar LN metastasis exhibits a similar prognosis to para-aortic LN metastasis spurred some investigators to raise questions regarding the stratification of splenic hilar LN as a extraperigastric LN and to suggest reclassifying splenic hilar LNs as a distant LN group [11]. In another study, the 5-year survival rate of patients with splenic hilar LN metastasis was significantly low at around 5%, even after curative total gastrectomy with D2 LN dissection, and was similar to that of patients undergoing R1 or R2 resection. Accordingly, the authors expressed doubts about the therapeutic impact of removing the splenic hilar LNs, suggesting that no benefit exists [14]. Meanwhile, however, other investigators reported that in the presence of curative surgery, the survival of hilar LN-positive patients did not differ from that of hilar LN-negative patients, and therefore, these investigators supported recommendations for D2 lymphadenectomy including splenic hilar LN dissection [19]. Similarly, in the present study, the 5-year overall survival of patients with splenic hilar LN metastasis was 24.1% and better than that in patients with distant metastasis. In addition, the survival of patients with splenic hilar LN metastasis was similar to patients with other extraperigastric LN metastasis, although the survival of patients with extraperigastric LN metastasis in this study remained poor. The poor survival rates in this study likely stem from the large number of metastatic LNs in the patients, who were stratified as having N3b disease according to current gastric cancer staging systems [20]. Moreover, around 73.6% (64 of 87 patients) of the patients experienced recurrence during follow-up after receiving curative treatment. The discrepancies between studies on the benefits of splenic hilar LN dissection probably stem from differences in the number of patients with LN #10 metastasis in the studies. Therefore, only small numbers of patients were included in the survival comparisons [10, 11, 14, 16]. In the current study, however, we included 87 patients with LN #10 metastasis without M1 disease, thereby allowing us to conduct a more comprehensive analysis.

As the prognosis of splenic hilar LN metastasis is still under debate, we sought to investigate whether classification of splenic hilar LNs as a regional LN group is appropriate. Thus, we conducted a unique comparison of splenic hilar LN metastasis with metastasis to other extraperigastric LNs, as well as distant metastasis, in terms of survival and recurrence patterns. In this comparison, the extraperigastric LN metastasis groups (LN #8a, #9, #11, and #12a, according to JGCA classification [7], which did not have hilar LN metastasis, showed similar long-term prognoses and recurrence patterns to patients with hilar LN metastasis. Although, there was no patient with metastasis to LN #10 without any extraperigastric LN metastasis, which might indicate splenic hilar LN was the farthest regional LN or boundary LN between the extraperigastric and distant LN, the survival between the patients with metastasis to LN #10 and those with metastasis to any extraperigastric but not to LN #10 metastasis did not significantly different. Additionally, the estimated therapeutic index value for splenic hilar LN dissection (3.5) was comparable to values for extraperigastric LN dissection (0.5–3.8). In support of these results, other studies have also reported similar therapeutic index values, ranging from 2.4–3.8, for LN #10 dissection of upper third gastric cancer [11, 21]. Taken together, these findings suggest that splenic hilar LNs should indeed continue to be classified as a regional LN group.

A recent Japanese multicenter prospective study (JCOG 0110) that examined splenectomy versus spleen-preservation during total gastrectomy for clinically advanced gastric cancer revealed no benefit of splenectomy in terms of operative safety and survival [22]; however, the study excluded tumors of the greater curvature, although these tumors are generally candidates for splenic hilar LN dissection. Thus, the study included clinically advanced gastric cancer patients who had little possibility of splenic hilar LN metastasis. Also, a large number of patients with pathologic T1 or N0 cancer was also included. Therefore, from these results alone, the actual therapeutic impact of splenic hilar LN dissection on advanced gastric cancer in patients with LN #10 involvement would be difficult to determine. In our study, however, we focused explicitly on the prognosis of splenic hilar LN metastasis, demonstrating acceptable survival and recurrence as well as a similar prognostic impact for splenic hilar LN dissection in comparison to dissection of metastases to other extraperigastric LNs.

Although current guidelines indicate D2 LN dissection, including splenic hilar LN dissection, for advanced (T2 or more advanced) gastric cancer during total gastrectomy, alterations of these indications to reflect tumor characteristics warrants discussion. As shown in this study, as well as in previous reports, significantly larger tumors, Borrmann type IV tumors, and advanced stage tumors involving the greater curvature or entire stomach were prominent in the hilar LN metastasis group [10–13, 15, 16, 23]. In addition, 96.5% (84/87) of patients who had hilar LN metastasis were staged IIIB or IIIC in the current study, while only 1.1% (3/267) of patients with hilar LN metastasis were staged IB to IIIA. A recent randomized controlled trial revealed no hilar LN metastasis in stage IIIA tumors or less advanced tumors that were staged according to the 6th TNM staging system, similar to our study [24]. Therefore, tumors not involving the greater curvature of the stomach that are small in size and of earlier stages may not warrant hilar LN dissection.

**Figure 4:**
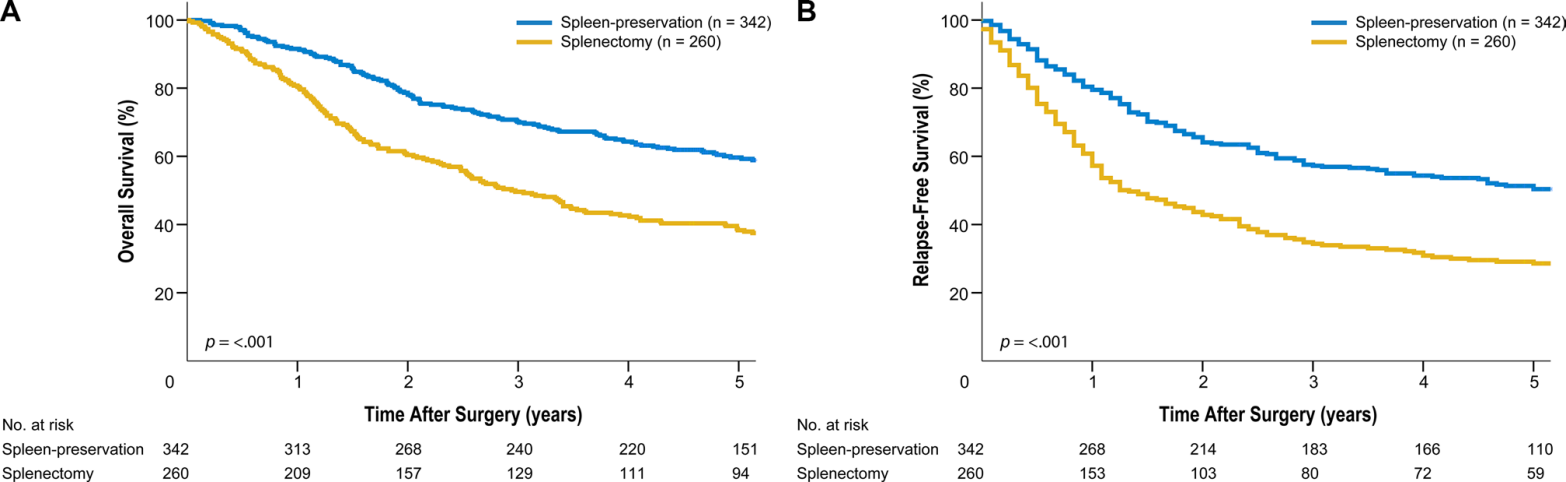
(Supplement) Kaplan-Meier survival curves for patients who underwent spleen-preserving hilar lymph node dissection and splenectomy. (**A**) Overall survival. (**B**) Relapse-free survival.

In our study, the indications for splenic hilar LN dissection likely varied among surgeons. In addition, the indications for either splenectomy or spleen-preserving hilar LN dissection could not be discerned, as this decision was made at the surgeon's discretion. The splenectomy group consisted of patients who had tumors of larger size at the greater curvature or circular location and were of advanced T classification/TNM stage. This group also included patients with a larger number of metastatic LNs (Table 4, Supplement). These characteristics reflected the poor survival outcomes in the splenectomy group (Figure 4, Supplement). Also, picking up LN #10 after spleen-preserving hilar LN dissection is difficult, although one surgeon who participated in the operation picked up each station just after the operation before sending it to the pathologist. As a potential consequence thereof, we obtained a significant difference in the number of retrieved LNs between splenectomy and spleen-preservation. Moreover, although we typically divide LN bearing tissue around the bifurcation of the upper or lower polar splenic artery from the main splenic artery to discriminate LN #10 from LN #11d, it can be difficult to divide them clearly. Additionally, there is the possibility that the actual number of retrieved LNs after spleen-preserving hilar LN dissection would be less, because posterior aspects of splenic vessels can be difficult to reach. This study also did not account for postoperative adjuvant chemotherapy, which may have an impact on patient survival. In general, patients with stage II or higher disease receive postoperative chemotherapy in accordance with our institution's standard postoperative care. Despite these limitations, this study included a large number of patients who underwent standard D2 LN dissection by experienced surgeons at a high-volume medical center. The results are significant because these findings encompass both therapeutic and prophylactic LN dissection for the treatment of proximal advanced gastric cancer. We discovered that patients with advanced gastric cancer with splenic hilar LN metastasis experience similar survival to those with other extraperigastric LN metastasis. These results suggest that splenic hilar LNs should be regarded as a regional LN group during total gastrectomy for the treatment of proximal advanced gastric cancer.

**Table 4:** (Supplement)

Variable	Spleen-preservation (*n* = 342)			Splenectomy (*n* = 260)			P*
	No. of Patients		%	No. of Patients		%	
Age, years							0.467
Mean		56.0			56.8		
SD		12.4			12.1		
Sex							0.377
Male	234		68.4	169		65.0	
Female	108		31.6	91		35.0	
Histologic type							0.634
Differentiated	86		25.1	61		23.5	
Undifferentiated	256		74.9	199		76.5	
Tumor size, cm							< 0.001
Mean		6.1			7.5		
SD		3.4			3.8		
Location							0.009
LC	172		50.3	116		44.6	
GC	30		8.8	36		13.8	
AW	51		14.9	39		15.0	
PW	84		24.6	54		20.8	
Circular	5		1.4	15		5.8	
Gross type							0.203
Borrmann I	21		6.2	17		6.5	
Borrmann II	63		18.4	61		23.5	
Borrmann III	179		52.3	111		42.7	
Borrmann IV	75		21.9	68		26.1	
Borrmann V	4		1.2	3		1.2	
LV invasion							0.033
negative	127		37.1	75		28.8	
positive	215		62.9	185		71.2	
Number of metastatic LNs							< 0.001
Mean		8.0			11.9		
SD		11.4			15.2		
Number of retrieved LNs							0.231
Mean		52.1			53.8		
SD		17.4			18.5		
LN #10 involvement							
positive	31		9.1	56		21.5	
negative	311		90.9	204		78.5	
Number of metastatic LNs at LN #10							< 0.001
Mean		0.2			0.6		
SD		0.6			1.7		
Number of retrieved LNs at LN #10							< 0.001
Mean		2.0			3.1		
SD		2.1			3.4		
T classification							< 0.001
T2	43		12.6	21		8.1	
T3	100		29.2	27		10.4	
T4a	192		56.1	192		73.8	
T4b	7		2.1	20		7.7	
N classification							0.092
N0	88		25.7	55		21.2	
N1	56		16.4	31		11.9	
N2	66		19.3	49		18.8	
N3	132		38.6	125		48.1	
TNM stage							< 0.001
Stage IB	26		7.6	11		4.2	
Stage IIA	47		13.8	8		3.1	
Stage IIB	48		14.0	43		16.5	
Stage IIIA	50		14.6	34		13.1	
Stage IIIB	63		18.4	49		18.9	
Stage IIIC	108		31.6	115		44.2	

Comparison of clinicopathologic factors between spleen-preserving hilar LN dissection and splenectomy patient groups.

## MATERIALS AND METHODS

**Figure 5:**
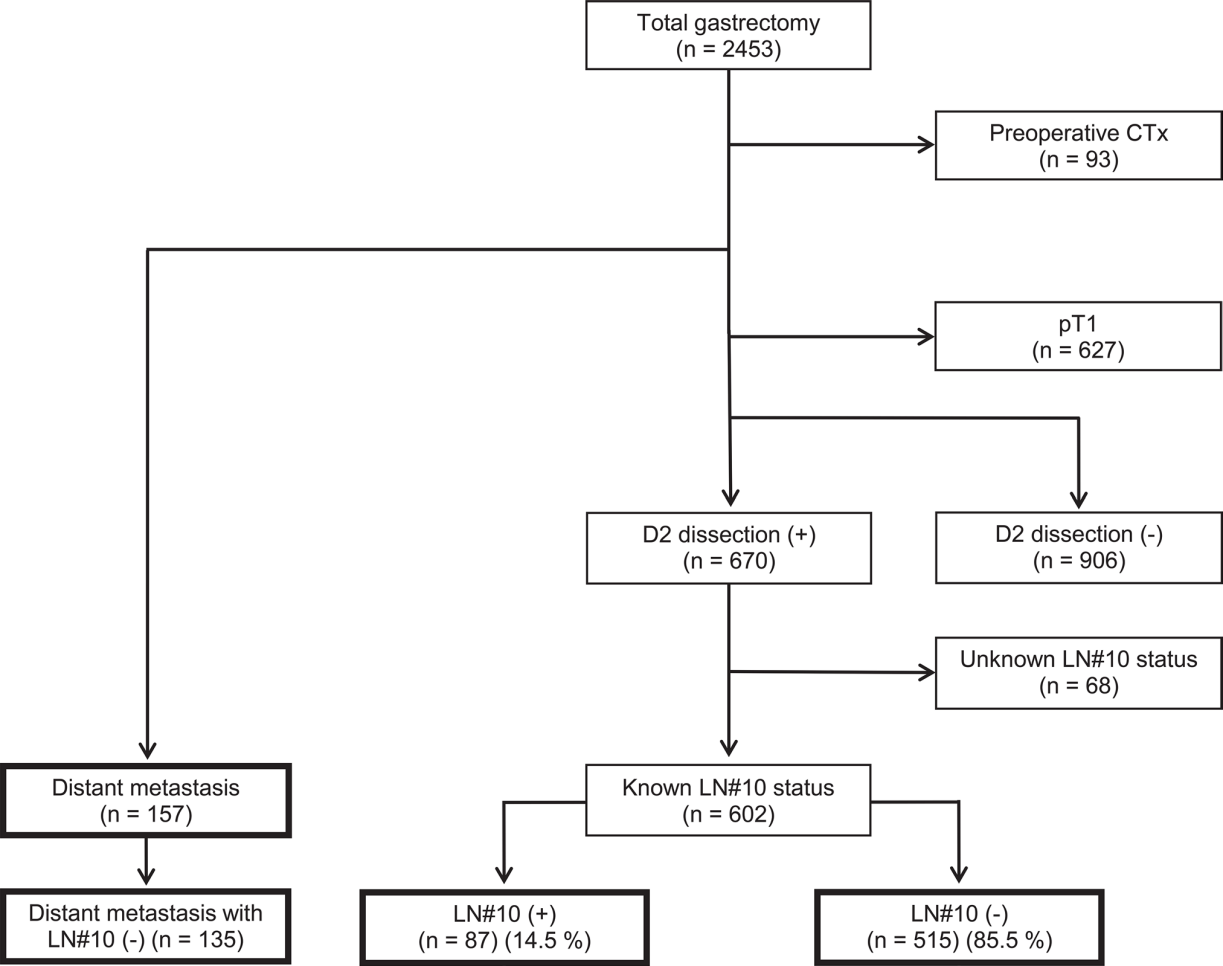
Study profile

Using a prospective database, we identified 2,453 patients who underwent total gastrectomy for gastric adenocarcinoma from 2000 to 2010 at Severance Hospital, Yonsei University Medical Center. Individual clinical and pathologic tumor characteristics, as well as short- and long-term patient outcomes, were retrieved. From the original cohort, 1,694 patients were excluded: 93 patients received preoperative chemotherapy with or without radiotherapy, 627 patients had T1 tumors, 906 patients did not undergo D2 LN dissection, and 68 patients had no information on splenic hilar LN metastasis (Figure 5). The final study population comprised 759 patients, among which 602 underwent curative total gastrectomy with D2 LN dissection. Of these patients, 87 out of 602 had splenic hilar LN metastasis and 157 had distant metastasis (M1) who received palliative total gastrectomy either splenic hilar lymph node dissection or not. Of all 157 patients with distant metastasis, 112 peritoneal metastases or Krukenberg tumors, 32 hematogenous metastases, six distant LNs metastases, and seven mixed metastases were included. Clinicopathologic characteristics, including age, sex, histologic differentiation, tumor size, location, gross type, lympho-vascular invasion, number of metastatic and retrieved LNs, and tumor-node-metastasis (TNM) stages, were included in the analyses. This retrospective study was approved by the Institutional Review Board of Severance Hospital, Yonsei University College of Medicine (4-2015-0951).

### Extent of lymphadenectomy

Total gastrectomy with D2 LN dissection was performed in accordance with the Japanese Gastric Cancer Association (JGCA) classification guidelines [7].

### Indications for splenectomy or spleen-preserving hilar LN dissection

At our institution, splenectomy during total gastrectomy with D2 LN dissection is indicated for gastric cancers with definite splenic hilar LN enlargement or direct invasion to the gastrosplenic ligament or the spleen. Spleen-preserving hilar LN dissection is indicated for gastric cancers with no serosal involvement and no evidence of gross LN metastasis along the splenic artery or splenic hilum during preoperative and intraoperative evaluation [17, 25]. We previously published our standard procedures for performing open or minimally invasive total gastrectomy with D2 LN dissection [25, 26].

### Indications for minimally invasive surgery

Patients with preoperative diagnosis of suspicious proper muscle invasion were indicated for total gastrectomy with D2 LN dissection, using either conventional laparoscopy or a robotic surgical system, since 2003 and 2005, respectively. Patients with evidence of serosal involvement or patients with any evidence of gross LN metastasis along the splenic artery or hilum were not generally considered to be indicated for minimally invasive surgery. All patients suitable for minimally invasive surgery were informed about procedures, including open gastrectomy and the risks and benefits of each procedure. All patients were allowed to select the type of operation that they would undergo, and all gave informed consent for the surgical method at the time of operation.

### Prognostic impact of splenic hilar LN metastasis

To evaluate the impact of splenic hilar LN metastasis on prognosis in gastric cancer patients, the survival of patients with metastasis to splenic hilar LNs (LN station #10) was compared to that of patients with metastasis to other extraperigastric LNs (e.g., LN stations #8a, #9, #11, or #12a) who did not concurrently have splenic hilar LN metastasis. For example, patients with metastasis to LN station #10 without metastasis to LN station #12a were compared to patients with metastasis to LN station #12a without metastasis to LN station #10. We also compared survival of the patients with metastasis to LN station #10 without metastasis to any extraperigastric LN to any extraperigastric LN station without LN station #10 metastasis. The overall survival curve of the splenic hilar metastasis group was compared to that of patients with distant metastasis (*n* = 157) and patients with distant metastasis without splenic hilar LN metastasis (*n* = 135).

### Estimated therapeutic index of splenic hilar LN dissection for gastric cancer

The estimated therapeutic index was first proposed as a means to estimate the benefit of removing an LN during surgery [21]. The index is calculated by multiplying the incidence of metastases by 5-year survival rates for individuals with nodal involvement at that specific station, regardless of nodal metastases to any other LN station.

### Patterns of recurrence

Patterns of recurrence were defined as locoregional, hematogenous, distant, peritoneal, and mixed, as in previous reports [27, 28].

### Statistical analysis

Clinicopathologic and short- and long-term operative results, including recurrence and survival, were analyzed using the Statistical Package for Social Sciences software, version 20.0 (IBM, Armonk, NY, USA). During the study period, patients were followed from the date of surgery until December 31, 2014 or their death. Overall survival was calculated from the date of operation to the last follow-up or death from any cause. Relapse-free survival was calculated from the date of operation to recurrence of gastric cancer or death from any cause. The date of recurrence was recorded as the day on which recurrence was confirmed by any imaging modality or tissue confirmation. Patients not experiencing the relevant end points were censored at last follow-up. Five-year overall survival and relapse-free survival were calculated using the Kaplan-Meier method, and differences in these rates between groups were examined using the log-rank test. Categorical and continuous variables were analyzed by the χ^2^ or Fisher's exact and Student's *t*-test, respectively. *P*-values < 0.05 (two-sided) were considered statistically significant.

## Abbreviations

LN: lymph node
JGCA: Japanese Gastric Cancer Association
JCOG: Japan Clinical Oncology Group
